# An analysis of equity in treatment of hip fractures for older patients with dementia in acute care hospitals: observational study using nationwide hospital claims data in Japan

**DOI:** 10.1186/s12913-020-05690-9

**Published:** 2020-09-04

**Authors:** Shinichi Tomioka, Megumi Rosenberg, Kiyohide Fushimi, Shinya Matsuda

**Affiliations:** 1 Department of Public Health and Health Policy, Graduate School of Biomedical & Health Sciences, Hiroshima University, 1-2-3 Kasumi, Hiroshima, 734-0037 Japan; 2 World Health Organization Centre for Health Development (WHO Kobe Centre), I.H.D. Centre Building 9th Floor, 1-5-1 Wakinohama-Kaigandori, Chuo-ku, Kobe, 651-0073 Japan; 3 Department of Health Policy and Informatics, Graduate School of Medicine, Tokyo Medical and Dental University, 1-5-45 Yushima, Bunkyo-ku, Tokyo, 113-8510 Japan; 4 Department of Preventive Medicine and Community Health, University of Occupational and Environmental Health, 1-1 Iseigaoka, Yahatanishi-ku, Kitakyushu, 807-8555 Japan

**Keywords:** Dementia, Ageism, Equity, Surgical treatment, Hip fracture, Hospital function

## Abstract

**Background:**

Globally, and particularly in countries with rapidly ageing populations like Japan, there are growing concerns over the heavy burden of ill health borne by older people, and the capacity of the health system to ensure their access to quality care. Older people with dementia may face even greater barriers to appropriate care in acute care settings. Yet, studies about the care quality for older patients with dementia in acute care settings are still few. The objective of this study is to assess whether dementia status is associated with poorer treatment by examining the association of a patient’s dementia status with the probability of receiving surgery and the waiting time until surgery for a hip fracture in acute care hospitals in Japan.

**Methods:**

All patients with closed hip fracture were extracted from the Diagnosis Procedure Combination (DPC) database between April 2014 and March 2018. After excluding complicated cases, we conducted regressions with multilevel models. We used two outcome measures: (i) whether the patient received a surgery or was treated by watchful waiting; and (ii) number of waiting days until surgery after admission.

**Results:**

Two hundred fourteen thousand six hundred one patients discharged from 1328 hospitals were identified. Among them, 159,173 patients received surgery. Both 80–89 year-olds (OR 0.87; 95% CI, 0.84, 0.90) and those 90 years old and above (OR 0.67; 95% CI, 0.65, 0.70) had significantly lower odds ratios for receiving surgery compared to 65–79 year-olds. Those with severe dementia had a significantly greater likelihood of receiving surgery compared to those without dementia (OR 1.21; 95% CI, 1.16, 1.25). Patients aged 90 years old and above had shorter waiting time for surgery (Coef. -0.06; 95% CI, − 0.11, − 0.01). Mild dementia did not have a statistically significant impact on the number of waiting days until surgery (*P* = 0.34), whereas severe dementia was associated with shorter waiting days (Coef. -0.08; 95% CI, − 0.12, − 0.03).

**Conclusions:**

These findings suggest physicians may be taking proactive measures to preserve physical function for those with severe dementia and to avoid prolonged hospitalization although there are no formal guidelines on prioritization for the aged and dementia patients.

## Background

Global population ageing is progressing rapidly. By 2050, one in six people in the world will be aged 65 years or over [1]. Improved survival beyond the age of 65 is fueling population ageing, putting increased financial pressure on the systems in place to support the older population, including healthcare. Now more than ever, countries need to ensure equity in healthcare with special attention to older people.

Older adults commonly perceive discrimination in healthcare settings due to their age [2, 3]. These perceptions are supported by empirical research findings of ageism at different levels of the healthcare system including age-biased clinical decision-making regarding diagnostics and treatments [4]. Negative attitudes of healthcare providers toward older patients are more commonly reported in acute health care settings, where targets and quick turnover are encouraged [5]. Studies suggest older people with dementia may face even greater barriers to appropriate care in acute care settings [6, 7].

Japan has the most aged population in the world. There are growing concerns over the heavy burden of ill health borne by older people, and the capacity of the country’s health system to ensure their access to quality care. Much attention is given to the increasing prevalence of dementia and its estimated societal cost, as they pose serious threats to the sustainability of the health and social care systems [8]. Yet, studies about the quality of care provided to the growing number of older patients living with dementia are still few [9, 10], and even fewer studies have considered the care they receive in acute care settings for medical conditions and co-morbidities other than dementia [11, 12].

The present study is one of the first studies in Japan to use hospital claims data to examine the receipt of acute care by older patients with dementia from an equity perspective. The objective of this study is to quantitatively assess whether dementia status is systematically associated with the likelihood of older patients receiving poorer treatment in acute care settings. Specifically, it will examine the association of a patient’s dementia status with the probability of receiving surgery and the waiting time until surgery for a hip fracture in acute care hospitals in Japan, controlling for other patient factors and contextual factors.

Hip fractures are a growing public health problem in Japan with the progression of population ageing. The estimated number of new hip fracture patients per year more than tripled from 53,200 new cases in 1987 to 175,700 in 2012 [13]. International consensus is that hip fractures among older people should be operated on within 48 h of hospital admission [14], although research shows that hip fracture surgery within 24 h could produce considerably better outcomes [15, 16]. On average across EU countries, more than three quarters (77%) of patients aged 65 and over admitted for a hip fracture were operated within 2 days in 2015, with most of them being treated either on the same day of their admission or the next day [14]. This is in accordance with a common guidance in Europe that hip fracture patients should receive surgery on the day of, or the day after, admission [17]. In Japan, similar guidance has not been issued by a national health authority, although the Japanese Orthopaedic Association recommends surgery within a week of admission [18]. Data from Japan show that the mean duration of preoperative hospital stay for hip fractures was 4.5 days, and the mean duration of hospitalization was 36.8 days in 2014. The long waiting time from hospitalization to surgery is reportedly due mainly to difficulties in securing operating rooms [19, 20].

Waiting time for surgery is a process indicator of the efficiency or quality of the health system response often used in international reporting [14]. Studies concerned with equity in healthcare focus on the differences in surgery waiting time by patient characteristics or by contextual factors such as urban or rural geography or hospital characteristics [21–23]. Many of them examine differences in waiting time for elective surgery by patient’s socioeconomic status. To the authors’ knowledge, only one study has been conducted in Japan to date which considered the effect of a patient’s dementia status on surgical delay for a hip fracture [20]. The study was conducted using data on 314 patients aged 60 years or above who were treated for hip fractures at one hospital between January 2006 and June 2012 and found no significant effect of dementia on surgical delay when controlling for other clinical and contextual factors. The present study will use a considerably larger database that covers over a thousand acute care hospitals across Japan.

## Methods

### Sources of data

The data were obtained from the Diagnosis Procedure Combination (DPC) database, a national administrative database commenced in 2003 with case-mix classification for the use of acute care inpatient reimbursement. Details of the DPC data are provided elsewhere [24, 25]. As of 2018, 1730 acute care hospitals out of 7134 all hospitals are reimbursed through the DPC [26]. Also, 69.2% of all general hospital beds are included in the DPC reporting system [27, 28].

In this study, we utilized 4 years of cross-sectional data from FF1 (or *Yoshiki* 1) of the DPC data covering the period of April 2014 to March 2018, which are the Japanese fiscal years of 2014 to 2017. In addition, we utilized detailed nationwide hospital data available from the Institute for Health Economics and Policy (IHEP) website [29] in order to append key hospital characteristics to each patient record.

### Study population

We selected all patients with a closed hip fracture (closed fracture of neck of femur, closed pertrochanteric fracture, and closed subtrochanteric fracture; ICD10 codes S72.00, S72.10 and S72.20, respectively). Although hip fracture is one of the most frequently encountered injuries in daily practice in Japan, because it is neither malignant nor an emergency, treatment varies widely depending on patient characteristics and environmental resources. Recent guidelines and studies recommend early surgical intervention [15, 16, 30–32].

We analyzed two outcome variables: (i) receipt of surgical operation (coded as 0 for no surgery and 1 for surgery performed) (i.e., not watching for spontaneous recovery), and (ii) number of waiting days until surgery following admission (coded as a continuous variable with a value of 0 assigned if the surgery was performed on the day of admission).

### Explanatory variables

The main explanatory variable of interest was the patient’s level of dementia and its impact on their functional ability as measured by the nationally standardized instrument used to assess the needs and eligibility for care under the long-term care insurance system. For the purposes of DPC data entry, the assessment is applied at the time of hospital admission to all patients 65 years old and older. There are six possible assessment outcomes: having no dementia (coded as 0); being on a scale of I to IV ranging from having some dementia but basically functionally independent (I) to requiring constant care due to severe symptoms or behavior and communication difficulties (IV); or having symptoms so severe that specialized medical care is required (coded M). For the present study, these were grouped into three categories: no dementia (coded as 0), mild dementia with little or no loss of function (coded as 1, comprising I and II above), and moderate to severe dementia with significant loss of function (coded as 2, comprising III, IV and M above). Analyses were also adjusted for age group, sex, fracture type (closed fracture of neck of femur, closed pertrochanteric fracture, closed subtrochanteric fracture), comorbidities (Charlson comorbidity index, groups 0–2), coma level, and ambulance use.

These conditions were routinely recorded in the DPC data except Charlson comorbidity index (CCI) which is calculated from patients’ comorbidities at the point of admission using Quan’s protocol [33]. While other conditions were recorded at the point of admission, fracture type could be modified during the patient’s hospital stay. Coma level was categorized into four consciousness depth levels using the Japan Coma Scale (JCS), which is routinely recorded in DPC data. Details of the JCS are described elsewhere [34]. Ambulance use was flagged when patients were transported by ambulance to reach the hospital. Ambulance use was included as a proxy for the level of emergency, which can also affect the probability of and time until surgery.

### Exclusions

We excluded all types of complicated cases from the study population in an attempt to equalize baseline conditions. We excluded patients who died within 24 h after admission, and those with co-existing severe trauma (e.g., brain bleeding), repeated surgery cases, or clinically complicated fractures which include bilateral, multiple, implant-related fractures or fracture with dislocation. We also excluded patients with multiple admissions within the 4-year study period, multiple surgeries within one admission, and patients who received surgery more than 180 days after admission. In addition, we excluded patients under 65 years old, because the DPC system does not require recording of dementia status for those younger patients. The impact of these exclusions was subsequently assessed by sensitivity analyses.

### Statistical models

For the first analysis of the probability of receiving surgical operation, we employed a multiple logistic regression model to obtain adjusted odds ratios (ORs) and 95% confidence intervals (CIs) associated with each explanatory variable using the entire study population. Then, a multiple linear regression model was applied to the subset of data on patients who received an surgery to obtain regression coefficients and 95% confidence intervals associated with each explanatory variable and the number of waiting days until the surgery. The explanatory variables described previously were all set as compositional factors, whereas hospital factors (i.e. city level and hospital function) were set as contextual factors. We built each model in four steps: 1) age and sex only, 2) age, sex and dementia level, 3) age, sex, dementia level and other patient clinical factors, and 4) the full model which included hospital and other contextual factors. Macro-level variance was calculated for each model using multilevel analysis. Details of multilevel analysis including the calculation of macro-level variance are described elsewhere [35]. Sensitivity analyses were also conducted for each of the exclusion criteria. All analyses were conducted using Stata 16.1.

## Results

### Sample extraction and characteristics

**Fig. 1:**
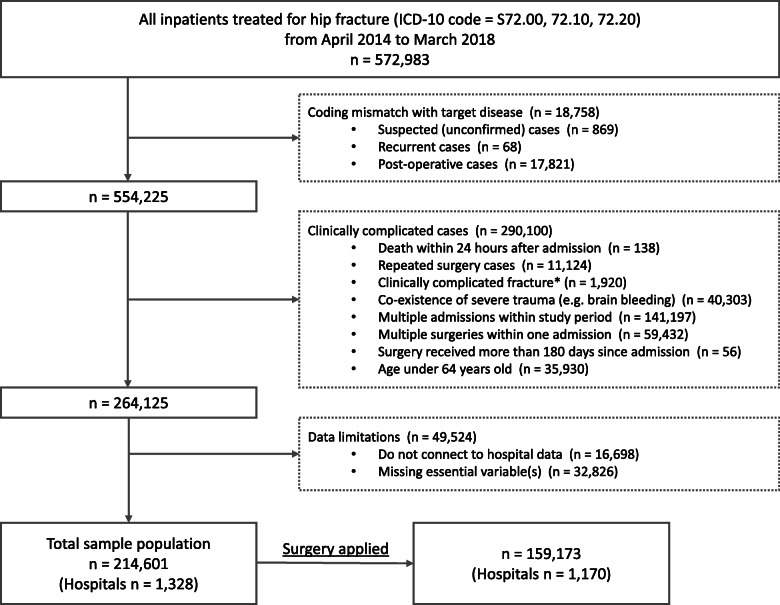
Study population extraction process. *ICD-10 International Classification of diseases, 10th revision. **Complicated fracture includes bilateral, multiple, implant related fractures or fracture with dislocation

From a total of 572,983 patients with a closed hip fracture recorded during the study period (April 2014–March 2018), 554,225 patients were extracted after confirming target disease name with ICD-10 codes. Secondly, clinically complicated cases were excluded, reducing the patient pool to 264,125. Thirdly, 49,524 patients were excluded due to limitations of missing values and hospital data. As a result, 214,601 patients discharged from 1328 hospitals were identified as the study population. Among them, 159,173 patients from 1170 hospitals received surgery, and thus, were included in the secondary analysis of waiting days until the surgery. The sample extraction process is summarized in Fig. 1.

Table 1 shows the baseline characteristics of the study sample and those who received a surgery. Females accounted for 77.9% of the total study sample, and the most common age group was 80–89 years old (49.2%). The number of those who were not diagnosed with dementia was 111,414 (51.9%), whereas 58,400 (27.2%) and 44,787 (20.9%) were diagnosed with mild and severe levels of dementia, respectively, in the total study sample. For the 159,173 patients who received a surgery, the mean number of waiting days was 3.66 (SD 3.72) days, with a median of 3 days. This indicates a longer waiting time than what is widely recommended in Europe, but is in accordance with relevant guidance in Japan [18].

**Table 1:** Baseline characteristics of the study population, total and surgery applied

	Total sample (%)		Surgery applied (%)	
	(*n* = 214,601)		(*n* = 159,173)	
**Individual factor**				
Sex				
Male	47,456	(22.1)	34,904	(21.9)
Female	167,145	(77.9)	124,269	(78.1)
Age Group				
65–79	57,608	(26.8)	44,528	(28.0)
80–89	105,505	(49.2)	78,333	(49.2)
Over 90	51,488	(24.0)	36,312	(22.8)
Dementia Level^a^				
0	111,414	(51.9)	84,424	(53.0)
1	58,400	(27.2)	42,187	(26.5)
2	44,787	(20.9)	32,562	(20.5)
Fructure Type				
Femoral neck	115,753	(53.9)	88,243	(55.4)
Trochanteric	95,946	(44.7)	69,229	(43.5)
Subtrochanteric	2900	(1.4)	1701	(1.1)
Charlson Index				
0	94,182	(43.9)	68,173	(42.8)
1	67,259	(31.3)	51,625	(32.4)
≥ 2	53,160	(24.8)	39,375	(24.7)
Coma Level^b^				
Alert	183,672	(85.6)	136,161	(85.5)
Level 1	29,890	(13.9)	22,301	(14.0)
Level 2	966	(0.5)	668	(0.4)
Level 3	73	(0.03)	43	(0.03)
Ambulance use				
No ambulance use	103,136	(48.1)	68,992	(43.3)
Ambulance use	111,465	(51.9)	90,181	(56.7)
**Hospital factor**				
City level^c^				
Designated city	62,667	(29.2)	47,388	(29.8)
Population ≥ 150,000	70,862	(33.0)	54,353	(34.2)
Population 80,000≤, ≤150,000	39,173	(18.3)	29,661	(18.6)
Population ≤ 80,000	41,899	(19.5)	27,771	(17.5)
Hospital function^d^				
University & advanced hospitals	5288	(2.5)	4263	(2.7)
Regional support hospital	52,802	(24.6)	44,825	(28.2)
Over 200 beds hospital	117,215	(54.6)	91,272	(57.3)
Under 200 beds hospital	39,296	(18.3)	18,813	(11.8)
**Length of stay factor**				
Discharge to				
Home	58,673	(27.3)	32,873	(20.7)
Recovery ward (same hosp.)	6318	(2.9)	3255	(2.04)
Other hospital	108,323	(50.5)	93,681	(58.9)
Long-term care facility	38,337	(17.9)	27,584	(17.3)
Death	2679	(1.3)	1605	(1.0)
Unknown	264	(0.1)	168	(0.1)

^a^Dementia level 1 and 2: represents “degree of independence in daily life for elderly people with dementia” criteria I-II and III-IV/M, respectively. ^b^Coma level refers to the Japan Coma Scale (JCS) which has four decisive levels of consciousness. ^c^Designated city has population over 500,000 and is designated by order of the Cabinet of Japan under Local Autonomy Law. ^d^Advanced hospitals include 6 national centers for cancer, circulation and global health. Regional support hospital has over 200 beds and meets requirements such as referral rate over 80% for outpatients

### Analysis 1: receipt of surgery

The results for the probability of receipt of surgery are shown in Table 2. In terms of age, both 80–89 year-olds (OR 0.87; 95% CI, 0.84, 0.90) and those 90 years old and above (OR 0.67; 95% CI, 0.65, 0.70) had significantly lower odds ratios for receiving surgery compared to 65–79 year-olds in the full model (model 4). With respect to dementia, in the full model, although patients with mild dementia were no more likely than those without dementia to receive surgery (OR 1.03; 95% CI, 1.00, 1.06), those with severe dementia had a significantly greater likelihood of receiving surgery compared to those without dementia (OR 1.21; 95% CI, 1.16, 1.25). Fracture type was also an important predictor of receiving surgery, with lower ORs observed for trochanteric (OR 0.79; 95% CI, 0.77, 0.81) and subtrochanteric fractures (OR 0.42; 95% CI, 0.38, 0.46). Patients with deeper coma levels were significantly less likely to receive a surgery (OR 0.56; 95% CI, 0.31, 1.01).

**Table 2:** Multivariate-adjusted odds ratios and 95% Confidence Intervals for application of surgery

***N*** = 214,601	model 1 (age, sex)				model 2 (Dementia level only)				model 3 (with clinical factors)				model 4 (with hospital factors)			
	OR	95% C.I.		*P* value	OR	95% C.I.		*P* value	OR	95% C.I.		*P* value	OR	95% C.I.		*P* value
**Compositional factor**																
Sex																
Male (reference)																
Female	1.15	1.12	1.19	< 0.001					1.16	1.12	1.19	< 0.001	1.16	1.12	1.19	< 0.001
Age Group																
65–79 (ref.)																
80–89	0.87	0.84	0.89	< 0.001					0.87	0.84	0.90	< 0.001	0.87	0.84	0.90	< 0.001
Over 90	0.67	0.64	0.69	< 0.001					0.67	0.65	0.70	< 0.001	0.67	0.65	0.70	< 0.001
Dementia Level^a^																
No dementia (ref.)																
Dementia level 1					0.94	0.91	0.97	< 0.001	1.03	1.00	1.06	0.08	1.03	1.00	1.06	0.06
Dementia level 2					1.06	1.02	1.09	< 0.001	1.20	1.16	1.25	< 0.001	1.21	1.16	1.25	< 0.001
Fx. Type																
Femoral neck (ref.)																
Trochanteric									0.79	0.77	0.81	< 0.001	0.79	0.77	0.81	< 0.001
Subtrochanteric									0.42	0.38	0.46	< 0.001	0.42	0.38	0.46	< 0.001
Charlson Index																
0 (ref.)																
1									1.18	1.15	1.22	< 0.001	1.18	1.15	1.22	< 0.001
≥ 2									1.00	0.97	1.03	0.96	1.00	0.97	1.03	0.93
Coma Level^b^																
Alert (ref.)																
Level 1									0.94	0.91	0.98	0.01	0.94	0.90	0.98	0.00
Level 2									0.67	0.56	0.80	< 0.001	0.67	0.56	0.80	< 0.001
Level 3									0.55	0.31	1.00	0.05	0.56	0.31	1.01	0.05
Ambulance Use																
No ambulance use (ref.)																
Ambulance use									1.37	1.33	1.40	< 0.001	1.36	1.33	1.40	< 0.001
**Contextual factor**																
City level^c^																
Designated city (ref.)																
Pop. ≥150,000													1.04	0.82	1.32	0.76
Pop. 80,000≤, ≤150,000													1.24	0.92	1.67	0.16
Pop. ≤80,000													0.97	0.74	1.26	0.80
Hosp. function^d^																
Under 200 beds hosp.(ref.)																
Over 200 beds hosp.													8.36	6.73	10.40	< 0.001
Regional support hosp.													15.07	11.13	20.42	< 0.001
University & advanced hosp.													10.90	7.05	16.83	< 0.001
**Macro-level variance (S.E.)**																
between hospitals (*n* = 1328)	4.18	(0.20)			4.20	(0.20)			4.04	(0.19)			2.83	(0.13)		

^a^Dementia level 1 and 2: represents “degree of independence in daily life for elderly people with dementia” criteria I-II and III-IV/M, respectively. ^b^Coma level refers to the Japan Coma Scale (JCS) which has four decisive levels of consciousness. ^c^Designated city has population over 500,000 and is designated by order of the Cabinet of Japan under Local Autonomy Law. ^d^Advanced hospitals include 6 national centers for cancer, circulation and global health. Regional support hospital has over 200 beds and meets requirements such as referral rate over 80% for outpatients

While a majority of compositional factors affected the probability of an older hip fracture patient receiving a surgery with statistical significance, the impact of contextual factors was rather negligible. In the full model, while city level did not significantly affect the probability of receiving a surgery, hospital function had a rather high impact, where the probability of receiving a surgery was highest in regional support hospitals (OR 15.07; 95% CI, 11.13, 20.42) followed by university and advanced hospitals (OR 10.90; 95% CI, 7.05, 16.83) and other types of hospitals with over 200 beds (OR 8.36; 95% CI, 6.73, 10.40) compared to other types of hospitals with under 200 beds, respectively. Reduction of macro-level variance from model 3 (4.04) to model 4 (2.83) also showed the impact of these contextual factors.

### Analysis 2: waiting days for surgery

The results for waiting time until surgery are shown in Table 3. Patients who were 90 years old and above had shorter waiting time (Coef. -0.06; 95% CI, − 0.11, − 0.01) compared to those aged 65 to 79, while the 80–89 year-old group did not (*P* = 0.10). In terms of dementia, similarly to the results for receipt of surgery, mild dementia did not have a statistically significant impact on the number of waiting days until surgery (*P* = 0.34), whereas severe dementia was associated with shorter waiting time (Coef. -0.08; 95% CI, − 0.12, − 0.03). Deeper coma levels incrementally lengthened waiting days; coefficients for coma level 2 and 3 were 0.26 (95% CI, 0.00, 0.51) and 1.18 (95% CI, 0.19, 2.17), respectively.

**Table 3:** Multivariate-adjusted coefficients and 95% CIs for waiting days for surgery with macro-level variance

***N*** = 159,173	model 1 (age, sex)				model 2 (Dementia level only)				model 3 (with clinical factors)				model 4 (with hospital factors)			
	coef.	95% C.I.		*P* value	coef.	95% C.I.		*P* value	coef.	95% C.I.		*P* value	coef.	95% C.I.		*P* value
**Compositional factor**																
Sex																
Male (reference)																
Female	−0.34	−0.38	−0.30	< 0.001					− 0.26	− 0.30	− 0.22	< 0.001	− 0.26	− 0.30	− 0.22	< 0.001
Age Group																
65–79 (ref.)																
80–89	−0.05	−0.09	−0.01	0.01					0.03	−0.01	0.07	0.10	0.03	−0.01	0.07	0.10
Over 90	−0.23	−0.28	− 0.19	< 0.001					−0.06	−0.11	− 0.01	0.02	− 0.06	−0.11	− 0.01	0.02
Dementia Level^a^																
No dementia (ref.)																
Dementia level 1					0.02	−0.02	0.06	0.28	0.02	−0.02	0.06	0.34	0.02	−0.02	0.06	0.34
Dementia level 2					−0.06	− 0.10	−0.01	0.01	−0.08	− 0.12	− 0.03	0.002	−0.08	− 0.12	−0.03	0.002
Fx. Type																
Femoral neck (ref.)																
Trochanteric									−0.72	−0.75	−0.68	< 0.001	−0.72	− 0.75	− 0.68	< 0.001
Subtrochanteric									−0.56	− 0.72	− 0.40	< 0.001	− 0.56	− 0.72	− 0.40	< 0.001
Charlson Index																
0 (ref.)																
1									0.39	0.35	0.43	< 0.001	0.39	0.35	0.43	< 0.001
≥ 2									0.72	0.68	0.77	< 0.001	0.72	0.68	0.77	0.93
Coma Level^b^																
Alert (ref.)																
Level 1									0.06	0.00	0.11	0.04	0.06	0.00	0.11	0.04
Level 2									0.26	0.00	0.51	0.05	0.26	0.00	0.51	0.05
Level 3									1.18	0.19	2.17	0.02	1.18	0.19	2.17	0.02
Ambulance Use																
No ambulance use (ref.)																
Ambulance use									0.16	0.12	0.19	< 0.001	0.16	0.12	0.19	< 0.001
**Contextual factor**																
City level^c^																
Designated city (ref.)																
Pop. ≥150,000													0.07	−0.21	0.34	0.62
Pop. 80,000≤, ≤150,000													−0.24	−0.58	0.10	0.16
Pop. ≤80,000													−0.27	−0.57	0.04	0.09
Hosp. function^d^																
Under 200 beds hosp.(ref.)																
Over 200 beds hosp.													−0.40	−0.66	−0.14	0.003
Regional support hosp.													−0.66	−1.00	−0.31	< 0.001
University & advanced hosp.													−0.01	−0.50	0.47	0.96
**Macro-level variance (S.E.)**																
between hospitals (*n* = 1170)	3.24	(0.15)			3.24	(0.15)			3.22	(0.15)			3.16	(0.15)		

^a^Dementia level 1 and 2: represents “degree of independence in daily life for elderly people with dementia” criteria I-II and III-IV/M, respectively. ^b^Coma level refers to the Japan Coma Scale (JCS) which has four decisive levels of consciousness. ^c^Designated city has population over 500,000 and is designated by order of the Cabinet of Japan under Local Autonomy Law. ^d^Advanced hospitals include 6 national centers for cancer, circulation and global health. Regional support hospital has over 200 beds and meets requirements such as referral rate over 80% for outpatients

Similarly with the results for receipt of surgery, city level was not associated with waiting time until surgery. However, two of the variables for hospital function had statistical significance, which were regional support hospital (Coef. -0.66; 95% CI, − 1.00, − 0.31) and other types of hospitals over 200 beds (Coef. -0.40, 95% CI, − 0.66, − 0.14). Unlike in the analysis of receiving surgery, macro-level variances between hospitals did not reduce from model 3 (3.22) to model 4 (3.16).

### Sensitivity analysis

Sensitivity analyses were conducted for each of potentially arbitrary exclusion criteria. First, we ran all models for patients with each of clinically complicated case (i.e. death within 24 h after admission, patients with co-existing severe trauma, repeated surgery cases, clinically complicated fractures, patients with multiple admissions within the 4-year study period, multiple surgeries within one admission). Then, we adjusted for patients waiting days from admission to surgery which was limited 180 days from admission in present study. We adjusted for waiting days from 30 days to 365 days. We confirmed the main results were invariant.

## Discussion

This study found no evidence of unfavorable treatment of patients with dementia for a hip fracture in acute care hospitals in Japan. On the contrary, the findings suggest that patients with severe dementia may be prioritized for surgery resulting in a greater likelihood of them receiving surgery. Furthermore, they may be given a shorter waiting time compared to patients without dementia or with only mild dementia who are otherwise similar in terms of clinical and contextual characteristics. Even patients with mild dementia are treated no differently from patients without dementia.

With regard to age, the study found that very old patients in their 80s and 90s are less likely to receive surgery compared to otherwise similar patients who are between the ages of 65 and 79. This result is concerning given that our analysis controlled for comorbidities and functional level. In other words, the observed difference cannot be explained by the possibility that the non-receipt of surgery among the older-old patients was clinically warranted, and thus ethical, due to contraindications or lower levels of functioning. However, for those who did receive surgery, the very old patients tend to have a shorter waiting time compared to the younger-old patients.

These findings suggest that although there are no formal guidelines on patient prioritization, physicians may be taking proactive measures to preserve physical function through surgery for those who are younger and for those with severe dementia. Once the decision to perform surgery is made, then it appears older patients and those with severe dementia are prioritized to avoid prolonged hospitalization for these patients for whom the consequences are likely to be negative. One study from Germany suggests conducting preoperative cognitive assessment (e.g. Mini Mental State Examination; MMSE) for very old patients arguing cognitive impairment is an important prognostic factor for the development of perioperative complications and the duration of the hospital stay [36]. In line with this suggestion, our findings indicate that physicians in Japan knowingly or unknowingly prioritize patients based on their cognitive function thereby helping to avoid undesirable outcomes.

However, the dataset we analyzed limits our understanding of the true causes of the observed patterns of treatment. We would like to think that the basis for the expedited surgery of patients with severe dementia and those who are very old is clinical benefit to the patient. However, it is also possible that hospitals are prioritizing and discharging these patients with complex needs who tend to have prolonged hospital stays, which can reduce turnover of hospital beds and reduce hospital revenue under prospective payment system. In fact, additional analysis from our study showed that the patients with dementia also had shorter lengths of hospital stay following surgery compared to patients with no dementia (Table 4). Qualitative research of the clinicians making these decisions would be informative. Whether the true driving force of this pattern is perceived benefit to the patient or financial incentive for the hospital, or both, the result for the patients with severe dementia and very old patients is that they have shorter waiting times until surgery, which in general is a good outcome. As these patients will require longer periods of recuperation and rehabilitation following discharge, early discharge should be followed by a supported discharge [5].

**Table 4:** Multivariate-adjusted coefficients and 95% CIs for length of hospital stay with macro-level variance

***N*** = 214,601	model 1 (all patients, ***N*** = 214,601)				model 2 (surgery only, ***N*** = 159,173)				model 3 (non-surgery only, ***N*** = 55,428)			
	coef.	95% C.I.		*P* value	coef.	95% C.I.		*P* value	coef.	95% C.I.		*P* value
**Compositional factor**												
Sex												
Male (reference)												
Female	−0.23	−0.44	− 0.02	0.04	− 0.60	− 0.77	− 0.44	< 0.001	0.31	− 0.34	0.96	0.35
Age Group												
65–79 (ref.)												
80–89	1.52	1.31	1.74	< 0.001	1.16	1.00	1.33	< 0.001	2.17	1.49	2.86	< 0.001
Over 90	0.85	0.58	1.11	< 0.001	0.92	0.72	1.13	< 0.001	1.32	0.52	2.12	< 0.001
Dementia Level^a^												
No dementia (ref.)												
Dementia level 1	0.67	0.44	0.89	< 0.001	0.21	0.04	0.39	0.02	1.96	1.28	2.63	< 0.001
Dementia level 2	−1.70	−1.96	− 1.44	< 0.001	−1.50	− 1.70	− 1.30	< 0.001	−0.56	− 1.34	0.22	0.16
Fx. Type												
Femoral neck (ref.)												
Trochanteric	0.24	0.06	0.42	0.01	−0.56	−0.70	− 0.42	< 0.001	2.38	1.84	2.93	< 0.001
Subtrochanteric	3.57	2.82	4.32	< 0.001	3.00	2.35	3.66	< 0.001	5.68	3.84	7.53	< 0.001
Charlson Index												
0 (ref.)												
1	1.03	0.82	1.24	< 0.001	0.97	0.81	1.13	< 0.001	1.39	0.73	2.06	< 0.001
≥ 2	2.32	2.09	2.55	< 0.001	2.46	2.28	2.64	< 0.001	2.55	1.84	3.26	< 0.001
Coma Level^b^												
Alert (ref.)												
Level 1	−0.94	−1.23	− 0.66	< 0.001	− 0.36	− 0.57	−0.14	< 0.001	− 1.43	−2.31	− 0.54	< 0.001
Level 2	− 0.42	−1.71	0.88	0.53	0.22	−0.82	1.25	0.68	0.21	−3.45	3.86	0.91
Level 3	−3.14	−7.81	1.53	0.19	0.72	−3.31	4.76	0.73	−8.37	−19.80	3.06	0.15
Ambulance Use												
No ambulance use (ref.)												
Ambulance use	1.05	0.87	1.24	< 0.001	1.94	1.80	2.08	< 0.001	−1.47	−2.10	−0.84	< 0.001
**Contextual factor**												
City level^c^												
Designated city (ref.)												
Pop. ≥150,000	−0.02	−1.58	1.53	0.98	−2.53	−3.78	−1.27	< 0.001	0.95	−1.00	2.90	0.34
Pop. 80,000≤, ≤150,000	0.25	−1.68	2.19	0.80	−1.96	−3.53	−0.39	0.02	0.68	−1.72	3.08	0.58
Pop. ≤80,000	1.51	−0.19	3.21	0.08	−0.15	−1.56	1.25	0.83	1.85	−0.25	3.96	0.09
Hosp. function^d^												
Under 200 beds hosp.(ref.)												
Over 200 beds hosp.	−7.87	−9.26	−6.47	< 0.001	−4.13	−5.37	−2.89	< 0.001	−8.42	−10.14	−6.70	< 0.001
Regional support hosp.	−12.04	− 13.98	− 10.09	< 0.001	−9.02	− 10.68	− 7.35	< 0.001	− 12.61	− 15.01	− 10.22	< 0.001
University & advanced hosp.	− 15.69	−18.50	− 12.87	< 0.001	− 10.41	− 12.75	−8.08	< 0.001	− 17.79	− 21.77	− 13.82	< 0.001
Discharge to												
Home (ref.)												
Recovery ward (same hosp.)	−22.33	−22.88	− 21.78	< 0.001	−15.66	−16.18	− 15.13	< 0.001	−27.83	− 29.06	− 26.60	< 0.001
Other hospital	−5.95	−6.19	−5.71	< 0.001	− 2.60	− 2.79	− 2.40	< 0.001	−15.82	− 16.60	− 15.04	< 0.001
Long-term care facility	−5.95	− 6.24	− 5.66	< 0.001	− 5.05	− 5.29	− 4.81	< 0.001	− 4.79	− 5.56	− 4.01	< 0.001
Death	1.46	0.66	2.26	< 0.001	− 1.93	−2.62	−1.25	< 0.001	8.47	6.48	10.46	< 0.001
Unknown	−2.75	−5.25	−0.24	0.03	−1.04	−3.13	1.06	0.33	−4.30	−10.74	2.14	0.19
**Macro-level variance (S.E.)**												
between hospitals	116.1	(5.30)			75.3	(3.70)			136.8	(7.27)		

^a^Dementia level 1 and 2: represents “degree of independence in daily life for elderly people with dementia” criteria I-II and III-IV/M, respectively. ^b^Coma level refers to the Japan Coma Scale (JCS) which has four decisive levels of consciousness. ^c^Designated city has population over 500,000 and is designated by order of the Cabinet of Japan under Local Autonomy Law. ^d^Advanced hospitals include 6 national centers for cancer, circulation and global health. Regional support hospital has over 200 beds and meets requirements such as referral rate over 80% for outpatients

This study also found that contextual factors, and especially the type or function of the hospital in which the patient received care had significant impact on the probability of the patient receiving surgery for their hip fracture and on their waiting time until surgery, above and beyond the effects of patient characteristics. The positive finding is that patients are not simply disadvantaged by their rural residence. Given that financial barriers to healthcare are minimized in Japan by the national health insurance system, geography, or rural residence, is one of the major concerns related to equity in healthcare. This study found that patients with comparable individual characteristics living in remote areas are just as likely to receive surgery as those living in urban areas without delay as long as they can seek care in high-functioning hospitals. Additional analysis showed a similar pattern in the length of hospital stay in which rural residence had no impact but the hospital’s functional level made a significant difference in the patient’s duration of hospitalization (Table 5).

**Table 5:** Relevant ICD10 codes

ICD10 code	Disease name	Variable name
S72.00	Closed fracture of neck of femur	Femoral neck (ref.)
S72.10	Closed pertrochanteric fracture	Trochanteric
S72.20	Closed subtrochanteric fracture	Subtrochanteric

### Limitations

The study population only included those patients experiencing hip fractures who received treatment in an acute care hospital which reports to the DPC data system. It did not include those patients who were admitted to a non-DPC-reporting hospital beds, which is 30.8% of all general hospital beds as of 2018, or who never received care in an acute care hospital. Thus, the generalizability of our findings is limited. In order to consider equity in access to care and treatment for hip fractures more broadly, we would need to examine the denominator of all older patients experiencing hip fractures in the community and take into factors such as physical access to an acute care hospital.

## Conclusion

We found hip fracture patients with severe dementia received surgery with a greater likelihood and with a shorter waiting time compared to patients without dementia or with only mild dementia. With regard to age, very old patients in their 80s and 90s are less likely to receive surgery compared to patients between the ages of 65 and 79. For those who did receive surgery, the very old patients tend to have a shorter waiting time. These findings suggest physicians providing acute care for hip fractures in hospitals in Japan may be taking proactive measures to preserve patient’s physical function and to avoid prolonged hospitalization based on their age or dementia level in the absence of formal guidelines on patient prioritization. In terms of contextual factors, rural residence in itself was not a disadvantage for these patients seeking care in acute care hospitals; rather, the functional level of the hospital in which they sought care was more likely to affect their likelihood of receiving surgery and the waiting time until surgery. Further study is required to elucidate the extent to which the observed treatment pattern serves the interests of the patient, the healthcare workers, and hospital business administration.

## Data Availability

The DPC dataset analyzed during the current study are not publicly available due to possible identification of personal information but are available from the corresponding author on reasonable request. Hospital data can be acquired from the IHEP website. https://www.ihep.jp/business/other/

## Abbreviations

CCI: Charlson Comorbidity Index
DPC: Diagnosis Procedure Combination
JCS: Japan Coma Scale
